# CAPE-*p*NO_2_ ameliorated diabetic nephropathy through regulating the Akt/NF-κB/ iNOS pathway in STZ-induced diabetic mice

**DOI:** 10.18632/oncotarget.23016

**Published:** 2017-12-07

**Authors:** Xiaoling Wang, Dejuan Li, Lu Fan, Qianhan Xiao, Hua Zuo, Zhubo Li

**Affiliations:** ^1^ College of Pharmaceutical Sciences, Southwest University, Chongqing 400716, China

**Keywords:** caffeic acid p-nitro phenethyl ester (CAPE-pNO_2_), diabetic nephropathy (DN), glomerular mesangial cells (GMCs), Akt/NF-κB/ iNOS pathway

## Abstract

Diabetic nephropathy (DN) is one of the most severe complications of diabetes mellitus. This study aimed to determine the effects and potential mechanism of caffeic acid para-nitro phenethyl ester (CAPE-*p*NO_2_), a derivative of caffeic acid phenethyl ester (CAPE), on DN; *In vivo*, intraperitoneal injections of streptozotocin (STZ) were used to induce diabetes in mice; then, the mice were intraperitoneally injected daily with CAPE or CAPE-*p*NO_2_ for 8 weeks. The mice were sacrificed, and blood samples and kidney tissues were collected to measure biological indexes. The results showed that CAPE and CAPE-*p*NO_2_ could lower serum creatinine, blood urea nitrogen, 24-h albumin excretion, malondialdehyde and myeloperoxidase levels and increase superoxide dismutase activity in diabetic mice. According to HE, PAS and Masson staining, these two compounds ameliorated structural changes and fibrosis in the kidneys. In addition, the immunohistochemical and western blot results showed that CAPE and CAPE-*p*NO_2_ inhibited inflammation through the Akt/NF-κB pathway and prevented renal fibrosis through the TGF-β/Smad pathway. *In vitro*, CAPE and CAPE-*p*NO_2_ inhibited glomerular mesangial cell (GMC) proliferation, arrested cell cycle progression and suppressed ROS generation. These compounds also inhibited ECM accumulation via regulating the TGF-β1, which was a similar effect to that of the NF-κB inhibitor PDTC. More importantly, CAPE and CAPE-*p*NO_2_ could up-regulate nitric oxide synthase expression in STZ-induced diabetic mice and HG-induced GMCs. CAPE-*p*NO_2_ had stronger effects than CAPE both *in vivo* and *in vitro*. These data suggest that CAPE-*p*NO_2_ ameliorated DN by suppressing oxidative stress, inflammation, and fibrosis via the Akt/NF-κB/ iNOS pathway.

## INTRODUCTION

Diabetic nephropathy (DN) is one of the major microvascular complications of diabetes and could be a common cause of chronic kidney disease (CKD) [1]. It has been reported that DN may lead to end-stage renal disease (ESRD) in both type 1 and type 2 diabetes mellitus; ESRD is becoming the most prevalent cause of morbidity and complications due to diabetes, and it is characterized by glomerular extracellular matrix (ECM) deposition, glomerular basement membrane thickening, glomerular mesangial cell (GMC) proliferation and metabolic abnormalities [2–5]. The pathogenesis of DN is complex and remains unclear. The mechanisms underlying the development of DN are generally considered to be oxidative stress, inflammation and fibrosis [6].

Oxidative stress is regarded as one of the major causes of the pathogenesis of DN, and oxidative stress caused by a chronic increase in reactive oxygen species (ROS) levels plays a pivotal role in kidney disease [7]. NOX4 (NADPH oxidase 4) plays a major role in ROS production; it is highly expressed in the kidneys and is an important homeostatic regulator in the pathogenesis of early-stage DN [8–10]. ROS generation can directly trigger a variety of stress-sensitive pathways and activate various downstream elements; and inflammation is a major response mediated by oxidative stress [11–12]. It has been reported that in DN, the overexpression of pro-inflammatory cytokines and chemokines, which include tumor necrosis factor-α (TNF-α), interleukin-1β (IL-1β) and interleukin-6 (IL-6), are associated with the NF-κB pathway in the inflammatory phase [13–16]. In addition, Akt is activated in streptozotocin (STZ)-induced diabetic mice and increases NF-κB transcription to promote DN progression; thus, NF-κB is a major downstream target of Akt [17, 18]. Activated NF-κB can also be transferred to the nucleus and induce the expression of its downstream target gene TGF-β1 to cause ECM accumulation, which leads to fibrosis in DN [19].

GMCs maintained the normal function of glomeruli, which belong to one of the five cell types of the kidneys. Their abnormal activation and phenotypic changes cause a series of lesions on the renal structure and alter renal function in DN [20–22]. High D-glucose (HG) promoted proliferation and hypertrophy in GMCs and induced high levels of ROS, which resulted in excessive ECM synthesis *in vitro*. The accumulation and increase in ECM components, such as collagen and fibronectin, in GMCs are hallmarks of the development of DN and contribute to renal fibrosis [23–27].

Nitric oxide (NO) is an important factor in renal hemodynamic regulation, and the role of NO in DN is complex. It is generally accepted that NO is overproduced in the early stage of DN and mediates glomerular hyperfiltration, but in the late stage of DN, NO levels are rapidly decreased and lead to glomerular sclerosis; these effects of NO are a double-edged sword in DN patients [28–30]. In animal models of CKD, blocking endothelial NO leads to an increase in microvascular disease, which is known to impair renal autoregulation [31]. Inducible nitric oxide synthase (iNOS) is the main unit of NO synthesis, and its levels are high during the early phase of wound healing; however, during the later phase of wound healing, paeoniflorin can promote iNOS synthesis in high glucose-induced macrophages through TLR2-dependent signaling pathways to alleviate the severity of DN [32, 33]. Therefore, NO concentrations and iNOS expression may be essential elements that need to be explored in DN.

Caffeic acid phenethyl ester (CAPE) has been identified as the main active component of *propolis* and has been reported to possess various pharmacological activities, including anti-oxidant, anti-inflammatory, anti-cancer, anti-fibrosis, anti-proliferation and anti-bacterial properties [34, 35]. It has been reported that CAPE attenuates fibrosis via inhibiting the TGF-β1/Smad3 pathway; in addition, CAPE can lower the expression levels of renal inflammatory chemokines and reversed the levels of SOD and MDA [36, 37]. In another study, it was found that treatment with CAPE could protect two murine cell lines from oxidative stress by inducing cellular anti-oxidant activity and preserving cellular morphology; CAPE is also an inhibitor of NF-κB that can suppress NF-κB transcription to lower the expression levels of inflammatory cytokines, such as IL-6, IL-β and TNF-α [38–41].

**Figure 1:**
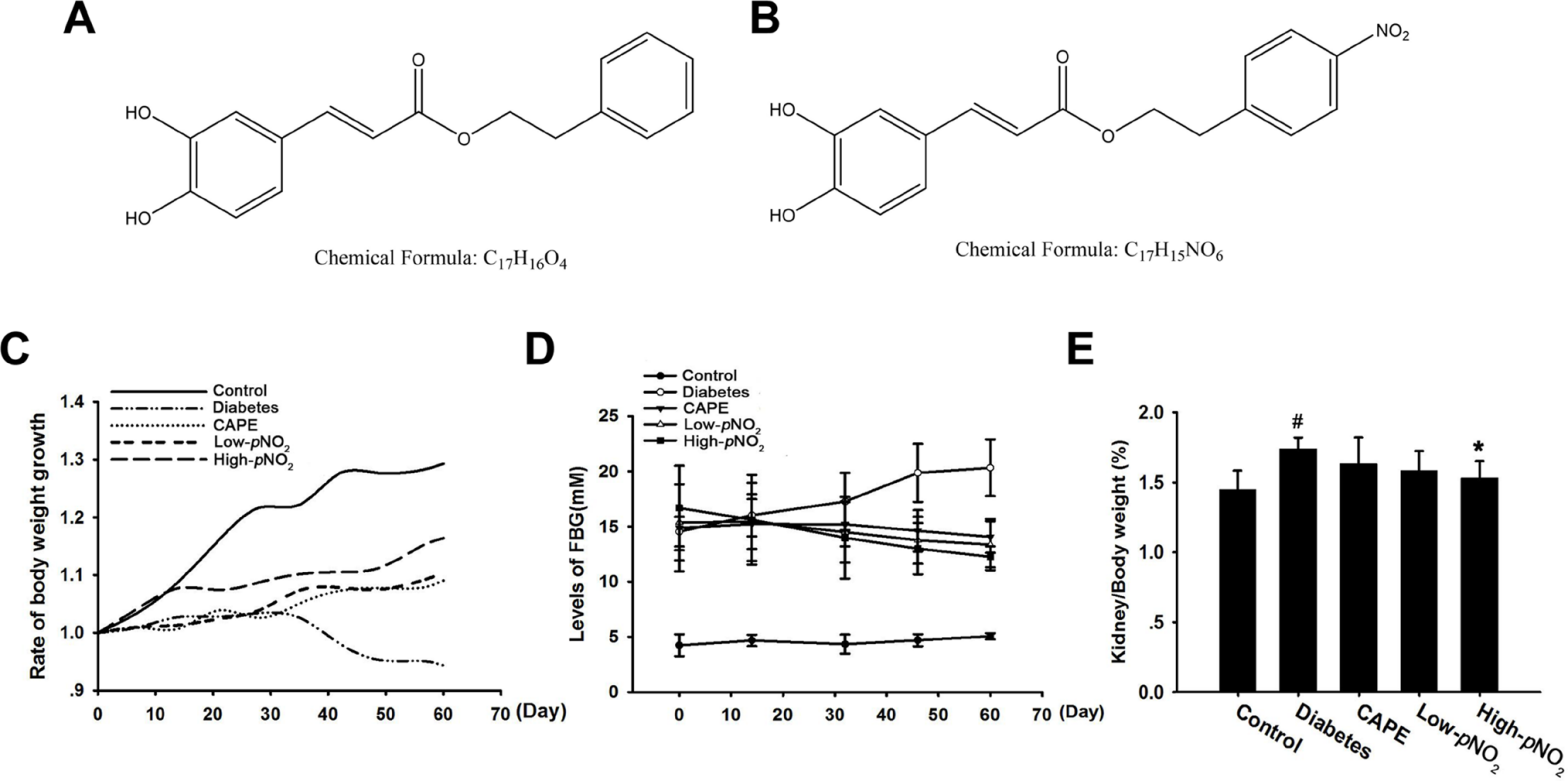
Effects of CAPE and CAPE-*p*NO_2_ treatment on biochemical indexes in diabetic mice (**A**) Chemical structure of CAPE. (**B**) Chemical structure of CAPE-*p*NO_2_. (**C**) The changed of the body weight during the 8 weeks. (**D**) The fast blood glucose. (**E**) The kidney/body weight. Data are expressed as mean ± SD, *n* ≥ 6. ^#^*p* < 0.05 vs. control group; ^*^p < 0.05 vs. diabetes group; ^+^*p* < 0.05 vs. CAPE group; ^&^*p* < 0.05 vs. Low-*p*NO_2_ group. Diabetes: the STZ-induced diabetic mice; CAPE: the STZ-induced diabetic mice treated with CAPE (20 μmol/kg/day); Low-*p*NO_2_: the STZ-induced diabetic mice treated with CAPE-*p*NO_2_ (20 μmol/kg/day); High-*p*NO_2:_ the STZ-induced diabetic mice treated with CAPE-*p*NO_2_ (40 μmol/kg/day).

In our previous study, caffeic acid para-nitro phenethyl ester (CAPE-*p*NO_2_), a derivative of CAPE, was designed and synthesized; CAPE-*p*NO_2_ had high anti-oxidative, anti-inflammatory and anti-colon cancer activities and protected against acute myocardial ischemia-reperfusion injury and promoted NO content in rat hearts [42, 43]. The structures of CAPE and CAPE-*p*NO_2_ are shown in Figure 1A and 1B.

In the present study, the differences between the effects of CAPE and CAPE-*p*NO_2_ on DN were examined. The objectives of this study were 1) to measure physiological and biochemical indexes in STZ-induced diabetic mice, 2) to examine the morphology changes by HE, PAS and Masson staining, and 3) to determine whether the better effects of CAPE-*p*NO_2_ on DN occurred via the Akt/NF-κB/iNOS pathway using *in vivo* and *in vitro* methods.

## RESULTS

### Effects of CAPE and CAPE-*p*NO_2_ treatment on biochemical indexes in diabetic mice

For eight weeks, body weights and FBG levels were recorded (Figure 1C, 1D). The body weights, FBG levels and kidney-to-body weight ratios (Figure 1E) in the diabetes group were 25.17 ± 0.94 g, 20.32 ± 2.56 mM and 1.73 ± 0.08%, respectively, while those in the control group were 42.67 ± 1.29 g, 5.06 ± 0.28 mM and 1.44 ± 0.14%, respectively. After treatment with CAPE or CAPE-*p*NO_2_ for 8 weeks, the body weights significantly increased to 30.15 ± 0.19 g, 32.35 ± 0.13 g and 32.89 ± 1.16 g in the CAPE, low CAPE-*p*NO_2_ and high CAPE-*p*NO_2_ groups, respectively (*p* < 0.05). Moreover, FBG levels were 14.06 ± 1.43 mM, 13.36 ± 2.31 mM and 12.66 ± 1.11 mM, and the kidney-to-body weight ratios were 1.63 ± 0.19%, 1.58 ± 0.14% and 1.52 ± 0.12% in the CAPE, low CAPE-*p*NO_2_ and high CAPE-*p*NO_2_ groups, respectively. FBG levels were significant decreased after treatment with CAPE or CAPE-*p*NO_2_ (*p* < 0.05), whereas the kidney-to-body weight ratios were significantly different in the high CAPE-*p*NO_2_ group only (*p* < 0.05).

BUN, sCr and 24-h Alb levels are shown in Table 1. After eight weeks of treatment, these levels were remarkably increased in the diabetes group compared to those in the control group (*p* < 0.05). However, treatment with CAPE and CAPE-*p*NO_2_ caused a significant reversal in these biochemical parameters (*p* < 0.05). TC and TG levels were also increased in the diabetic mice, but CAPE and CAPE-*p*NO_2_ treatment reversed these levels (*p* < 0.05). There was a significant difference between CAPE and CAPE-*p*NO_2_ treatment for 24-h Alb and TG levels (*p* < 0.05) but no differences for BUN, sCr and TC levels.

**Table 1:** Effects of CAPE and CAPE-*p*NO_2_ treatment on blood urea nitrogen, serum creatinine, 24 h albumin, triglyceride and cholesterol in diabetic mice

	Control Group	Diabetes Group	Diabetes + Treatment Group		
			CAPE(20 μmol/kg)	CAPE-*p*NO_2_(20 μmol/kg)	CAPE-*p*NO_2_(40 μmol/kg)
sCr (μmol/L)	46.26 ± 9.09	95.96 ± 2.74^#^	59.73 ± 1.98^*^	54.38 ± 2.04^*^	43.42 ± 4.79^*&^
BUN (mmol/L)	8.02 ± 0.42	15.16 ± 1.55^#^	12.12 ± 0.24^*^	11.12 ± 1.23^*^	10.67 ± 0.98^*^
Alb (mg/24h)	1.31 ± 0.03	18.19 ± 0.44^#^	12.16 ± 0.53^*^	10.15 ± 0.42^*+^	6.68 ± 0.31^*&^
TC (mmol/L)	3.22 ± 0.11	4.44 ± 0.06^#^	3.43 ± 0.20^*^	3.32 ± 0.16^*^	3.09 ± 0.05^*&^
TG (mmol/L)	0.77 ± 0.02	2.73 ± 0.10^#^	1.87 ± 0.39^*^	1.73 ± 0.05^*+^	1.36 ± 0.03^*&^

Data are expressed as mean ± SD, *n* ≥ 6. ^#^*p* < 0.05 vs. control group; ^*^p < 0.05 vs. diabetes group; ^+^*p* < 0.05 vs. CAPE group; ^&^*p* < 0.05 vs. Low-CAPE-*p*NO_2_ group.

### Effects of CAPE and CAPE-*p*NO_2_ treatment on oxidative stress in diabetic mice

**Figure 2:**
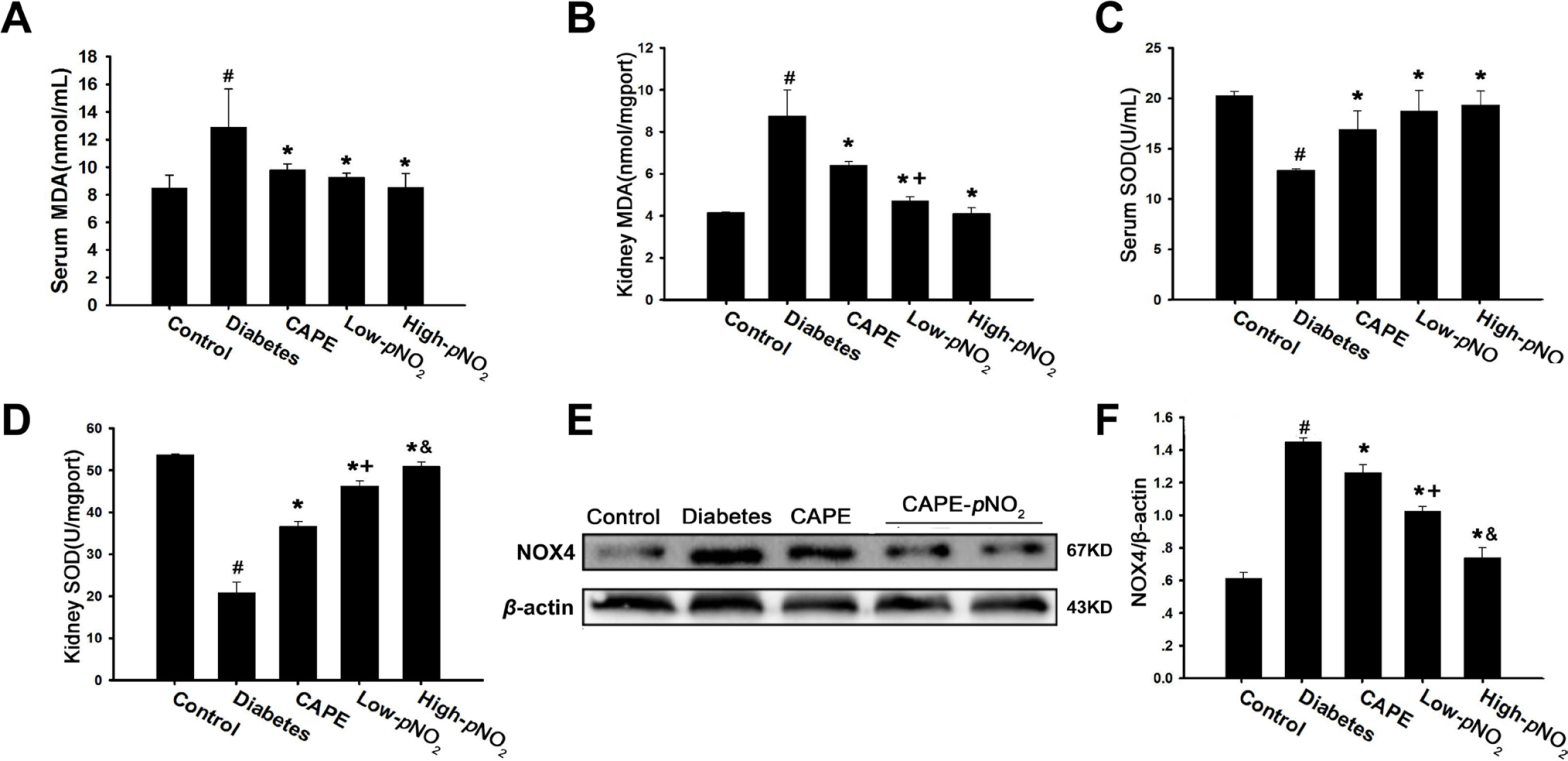
Effects of CAPE and CAPE-*p*NO_2_ treatment on oxidative stress in diabetic mice (**A**, **B**) CAPE and CAPE-*p*NO_2_ treatment elevate the SOD activity in serum and kidney tissue. (**C**, **D**) CAPE and CAPE-*p*NO_2_ treatment decrease the MDA level in serum and kidney tissue. (**E**, **F**) The expression of NOX4 was assessed by western blot analysis. Data are expressed as mean ± SD, *n* ≥ 6. ^#^*p* < 0.05 vs. control group; ^*^p < 0.05 vs. diabetes group; ^+^*p* < 0.05 vs. CAPE group; ^&^*p* < 0.05 vs. Low-*p*NO_2_ group. Diabetes: the STZ-induced diabetic mice; CAPE: the STZ-induced diabetic mice treated with CAPE (20 μmol/kg/day); Low-*p*NO_2_: the STZ-induced diabetic mice treated with CAPE-*p*NO_2_ (20 μmol/kg/day); High-*p*NO_2:_ the STZ-induced diabetic mice treated with CAPE-*p*NO_2_ (40 μmol/kg/day).

The levels of MDA and SOD were measured to estimate the anti-oxidative effects of CAPE and CAPE-*p*NO_2_. As shown in Figure 2A–2D, MDA levels were increased, and SOD activity was inhibited in diabetic mice (*p* < 0.05). Treatment with CAPE or CAPE-*p*NO_2_ remarkably suppressed the MDA levels and increased SOD activity (*p* < 0.05), and the effects of CAPE-*p*NO_2_ were much better than those of CAPE in the kidney tissues (*p* < 0.05); however, there was no significant difference in the effects on serum.

NOX4 expression levels were increased 1.4-fold in the diabetes group compared to those in the control group (*p* < 0.05) as shown in Figure 2E and 2F. However, NOX4 expression levels in the CAPE, low CAPE-*p*NO_2_ and high CAPE-*p*NO_2_ groups were decreased 1.2-, 1.0- and 0.7- fold (*p* < 0.05). In addition, there were significant differences between CAPE and CAPE-*p*NO_2_ treatment (*p* < 0.05).

### Effects of CAPE and CAPE-*p*NO_2_ treatment on the kidney structure and fibrosis in diabetic mice

**Figure 3:**
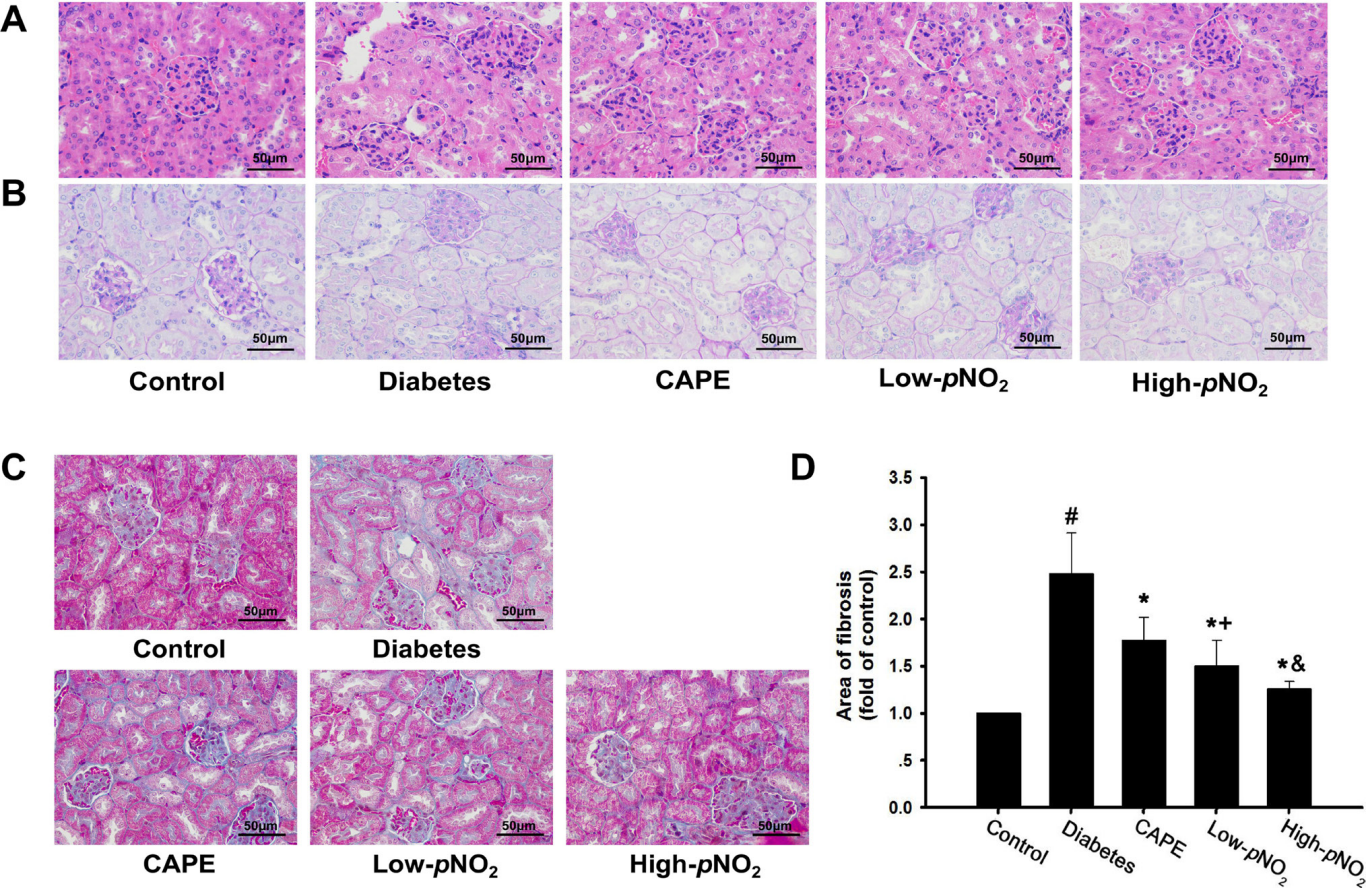
Effects of CAPE and CAPE-*p*NO_2_ treatment on the kidney structure and fibrosis in diabetic mice (**A**, **B**) Paraffin- embedded kidney tissue stained with H&E and PAS staining were observed under light microscope (400x, bar = 50 μm). (**C**, **D**) Paraffin-embedded kidney tissue was stained with Masson staining, the blue area represents the fibrosis in kidney tissue (400x, bar = 50 μm). Data are expressed as mean ± SD, *n* ≥ 6. ^#^*p* < 0.05 vs. control group; ^*^p < 0.05 vs. diabetes group; ^+^*p* < 0.05 vs. CAPE group; ^&^*p* < 0.05 vs. Low-*p*NO_2_ group. Diabetes: the STZ-induced diabetic mice; CAPE: the STZ-induced diabetic mice treated with CAPE (20 μmol/kg/day); Low-*p*NO_2_: the STZ-induced diabetic mice treated with CAPE-*p*NO_2_ (20 μmol/kg/day); High-*p*NO_2:_ the STZ-induced diabetic mice treated with CAPE-*p*NO_2_ (40 μmol/kg/day).

According to HE and PAS staining (Figure 3A, 3B), the control group had intact glomeruli and tubule morphology, whereas the diabetes group had basement membrane thickening of the renal tubules and glomerular hypertrophy. After treatment with CAPE and CAPE-*p*NO_2_ for 8 weeks, the renal structures were improved compared with those in diabetic mice.

For Masson staining, the blue-colored areas of the kidney tissues represent collagen, which indicates fibrosis (Figure 3C, 3D). The deposition of collagen in the glomerular and tubular lumen was 2.5-fold higher in the diabetes group than in the control group (*p* < 0.05), whereas that in the CAPE, low CAPE-*p*NO_2_ and high CAPE-*p*NO_2_ groups was decreased 1.8-, 1.5- and 1.3-fold, respectively.

### CAPE and CAPE-*p*NO_2_ inhibited inflammation through the Akt/NF-κB pathway in diabetic mice

**Figure 4:**
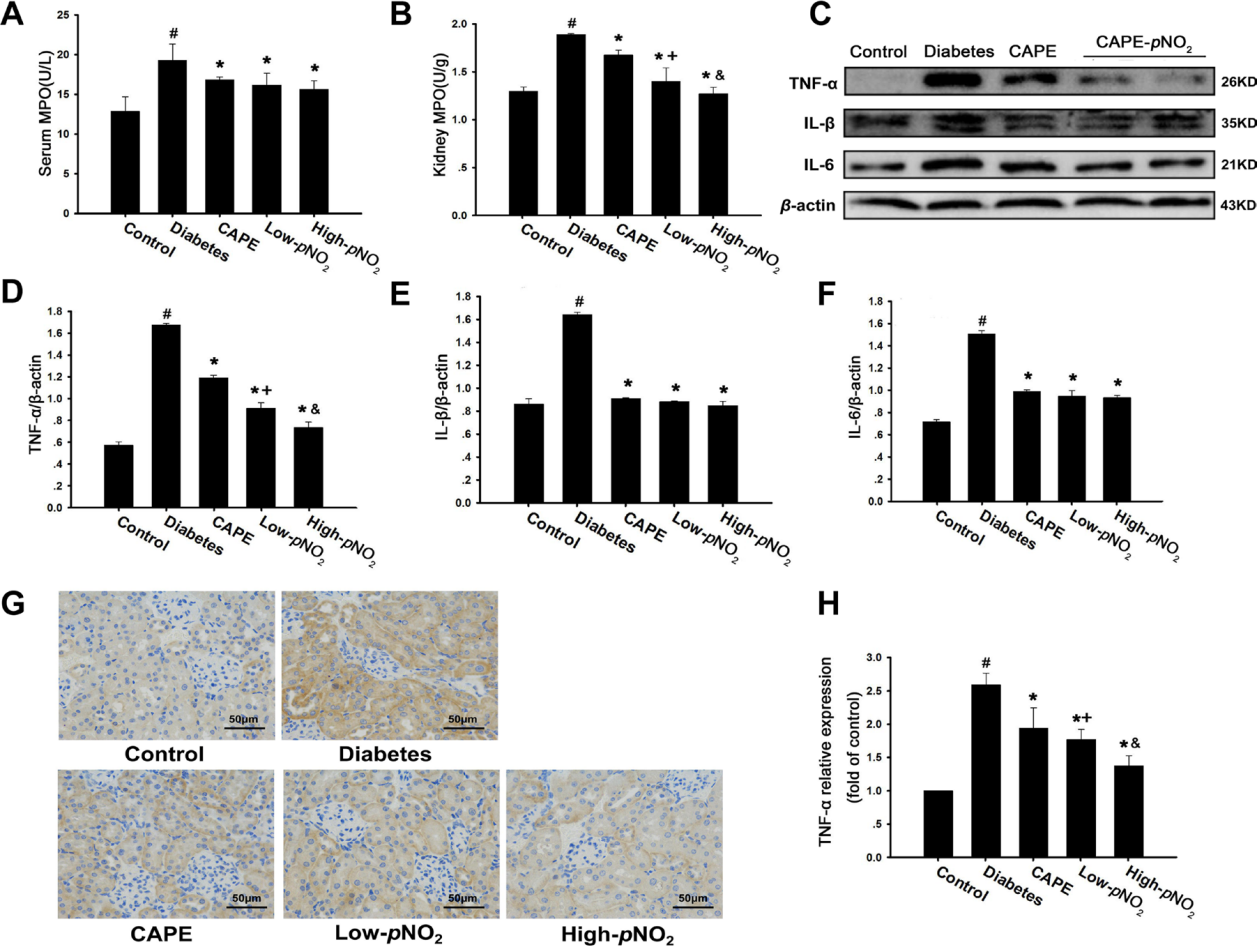
CAPE and CAPE-*p*NO_2_ inhibited inflammatory cytokines expression in diabetic mice (**A**, **B**) CAPE and CAPE-*p*NO_2_ treatment on MPO level in serum and kidney tissue. (**C**–**F**) The expressions of IL-6, tumor necrosis factor α (TNF-α) and interleukin-1β (IL-1β) were assessed by western blot analysis. (**G**, **H**) the immunohistochemical analysis of tumor necrosis factor α (TNF-α) activity in kidney sections. Data are expressed as mean ± SD, *n* ≥ 6. ^#^*p* < 0.05 vs. control group; ^*^p < 0.05 vs. diabetes group; ^+^*p* < 0.05 vs. CAPE group; ^&^*p* < 0.05 vs. Low-*p*NO_2_ group. Diabetes: the STZ-induced diabetic mice; CAPE: the STZ-induced diabetic mice treated with CAPE (20 μmol/kg/day); Low-*p*NO_2_: the STZ-induced diabetic mice treated with CAPE-*p*NO_2_ (20 μmol/kg/day); High-*p*NO_2:_ the STZ-induced diabetic mice treated with CAPE-*p*NO_2_ (40 μmol/kg/day).

As shown in Figure 4A and 4B, MPO levels were significantly increased in diabetic mice (*p* < 0.05). CAPE and CAPE-*p*NO_2_ injections decreased the MPO levels in the serum and kidney tissues (*p* < 0.05). The expression levels of TNF-α, IL-1β and IL-6, which are pro-inflammatory cytokines and chemokines, were increased in diabetic mice according to western blot and immunohistochemical assays (*p* < 0.05). Treatment with CAPE and CAPE-*p*NO_2_ down-regulated their expression levels significantly (*p* < 0.05). In particular, the effects of CAPE-*p*NO_2_ were much greater than those of CAPE on TNF-α (*p* < 0.05), but there were no significant differences in the levels of IL-1β and IL-6 (Figure 4C–4H).

**Figure 5:**
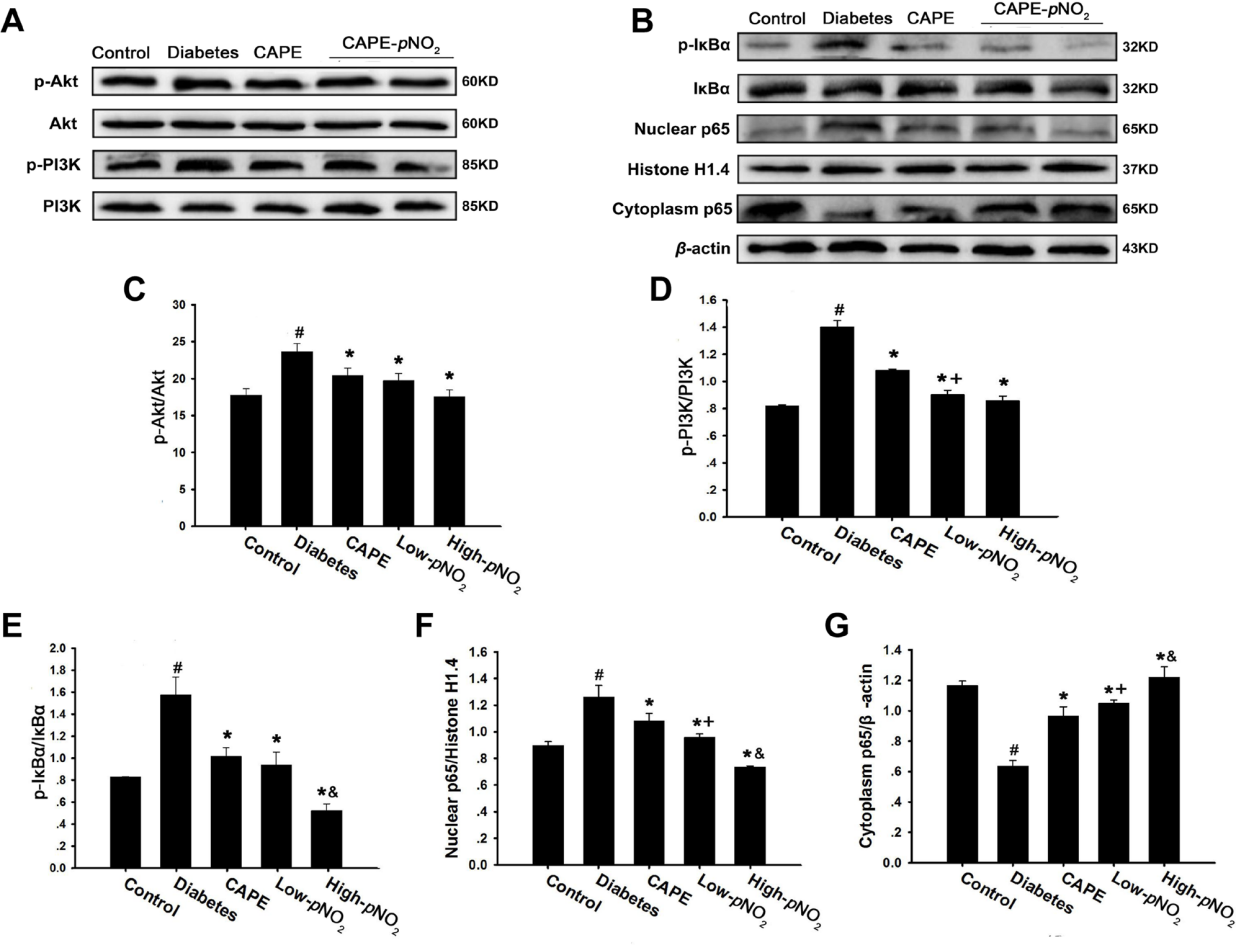
CAPE and CAPE-*p*NO_2_ inhibited inflammation through the Akt/NF-κB pathway in diabetic mice (**A**–**G**) The expressions of PI3K, p-PI3K, Akt, p-Akt, p-IκBα, IκBα and p65 in nuclear and cytoplasm were assessed by western blot. Data are expressed as mean ± SD, *n* ≥ 6. ^#^*p* < 0.05 vs. control group; ^*^p < 0.05 vs. diabetes group; ^+^*p* < 0.05 vs. CAPE group; ^&^*p* < 0.05 vs. Low-*p*NO_2_ group. Diabetes: the STZ-induced diabetic mice; CAPE: the STZ-induced diabetic mice treated with CAPE (20 μmol/kg/day); Low-*p*NO_2_: the STZ-induced diabetic mice treated with CAPE-*p*NO_2_ (20 μmol/kg/day); High-*p*NO_2:_ the STZ-induced diabetic mice treated with CAPE-*p*NO_2_ (40 μmol/kg/day).

There is evidence indicating that TNF-α, IL-1β and IL-6 expression can be up-regulated by the NF-κB pathway. As shown in Figure 5A–5G, the expression levels of nuclear p65, p-IκBα, p-Akt and p-PI3K in the NF-κB pathway were all increased, whereas the expression levels of cytoplasm p65 were decreased in the diabetes group (*p* < 0.05). However, treatment with CAPE and CAPE-*p*NO_2_ decreased nuclear p65, p-IκBα, p-Akt and p-PI3K expression levels and increased the expression levels of cytoplasm p65 (*p* < 0.05). In addition, CAPE-*p*NO_2_ was more effective than CAPE, particularly its effects on p-PI3K and p65 (*p* < 0.05). These results indicated that the inhibition of inflammation caused by CAPE and CAPE-*p*NO_2_ may occur through the Akt/NF-κB pathway in diabetic mice and that the effects of CAPE-*p*NO_2_ were stronger.

### CAPE and CAPE-*p*NO_2_ prevented renal fibrosis through the TGF-β/Smad pathway in diabetic mice

**Figure 6:**
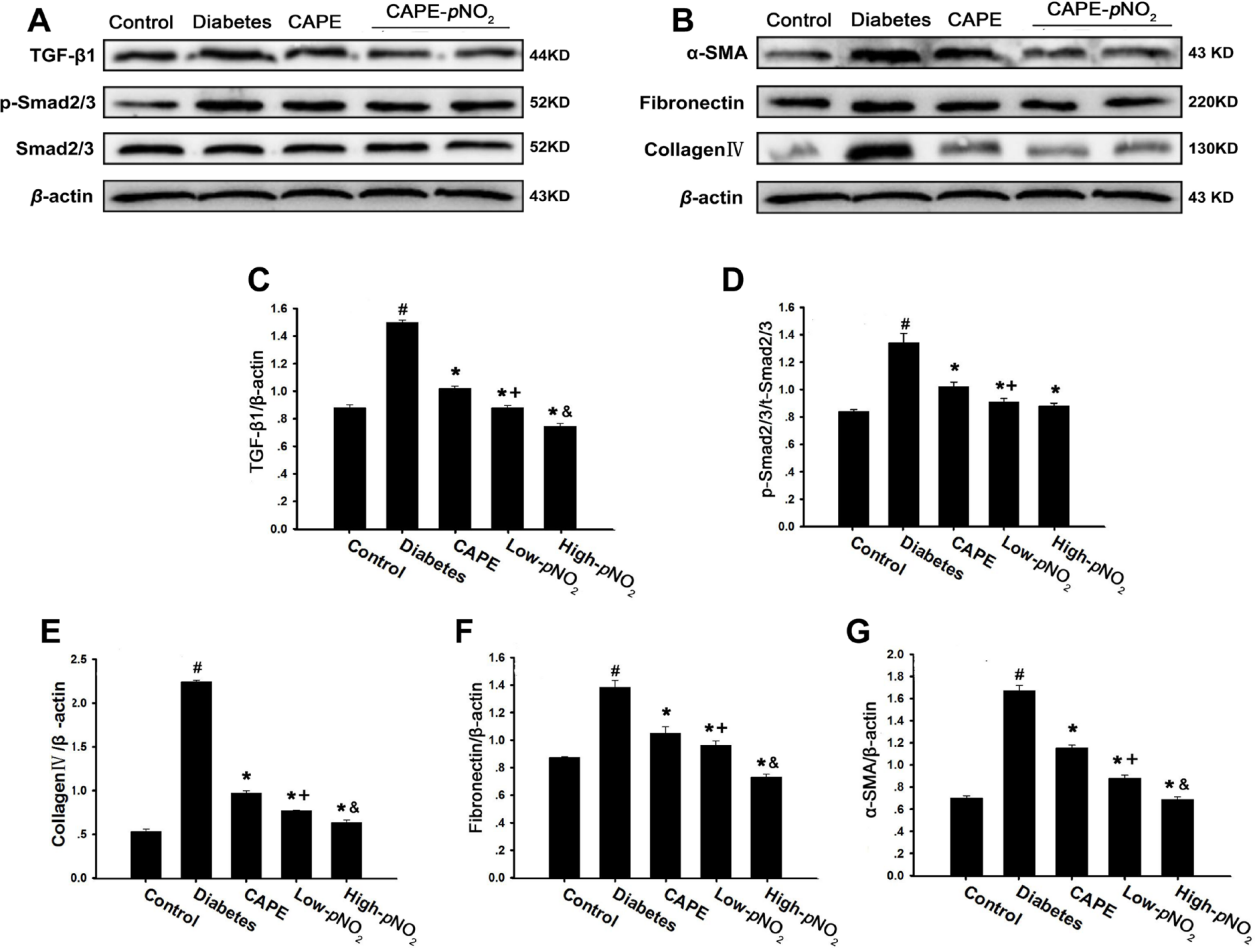
CAPE and CAPE-*p*NO_2_ prevented renal fibrosis through the TGF-β/Smad pathway in diabetic mice (**A**–**G**) The expressions of TGF-β1, collagenIV, fibronectin, α-SMA, p-Smad2/3 and Smad2/3 were assessed by western blot analysis. Data are expressed as mean ± SD, *n* ≥ 6. ^#^*p* < 0.05 vs. control group; ^*^p < 0.05 vs. diabetes group; ^+^*p* < 0.05 vs. CAPE group; ^&^*p* < 0.05 vs. Low-*p*NO_2_ group. Diabetes: the STZ-induced diabetic mice; CAPE: the STZ-induced diabetic mice treated with CAPE (20 μmol/kg/day); Low-*p*NO_2_: the STZ-induced diabetic mice treated with CAPE-*p*NO_2_ (20 μmol/kg/day); High-*p*NO_2:_ the STZ-induced diabetic mice treated with CAPE-*p*NO_2_ (40 μmol/kg/day).

The results of Masson staining indicated that CAPE and CAPE-*p*NO_2_ treatment could rescue renal fibrosis in diabetic mice. As shown in Figure 6A–6G, the expression levels of TGF-β1, collagen IV, fibronectin, α-SMA and p-Smad2/3, which are associated with the TGF-β/Smad pathway, were examined by western blot. The expression levels of all these proteins were obviously up-regulated in the diabetes group (*p* < 0.05). However, the expression levels of these proteins were suppressed by CAPE and CAPE-*p*NO_2_ treatment (*p* < 0.05), and the inhibition by CAPE-*p*NO_2_ was more powerful than that of CAPE (*p* < 0.05).

### CAPE and CAPE-*p*NO_2_ increased NO content via regulating iNOS expression in diabetic mice

**Figure 7:**
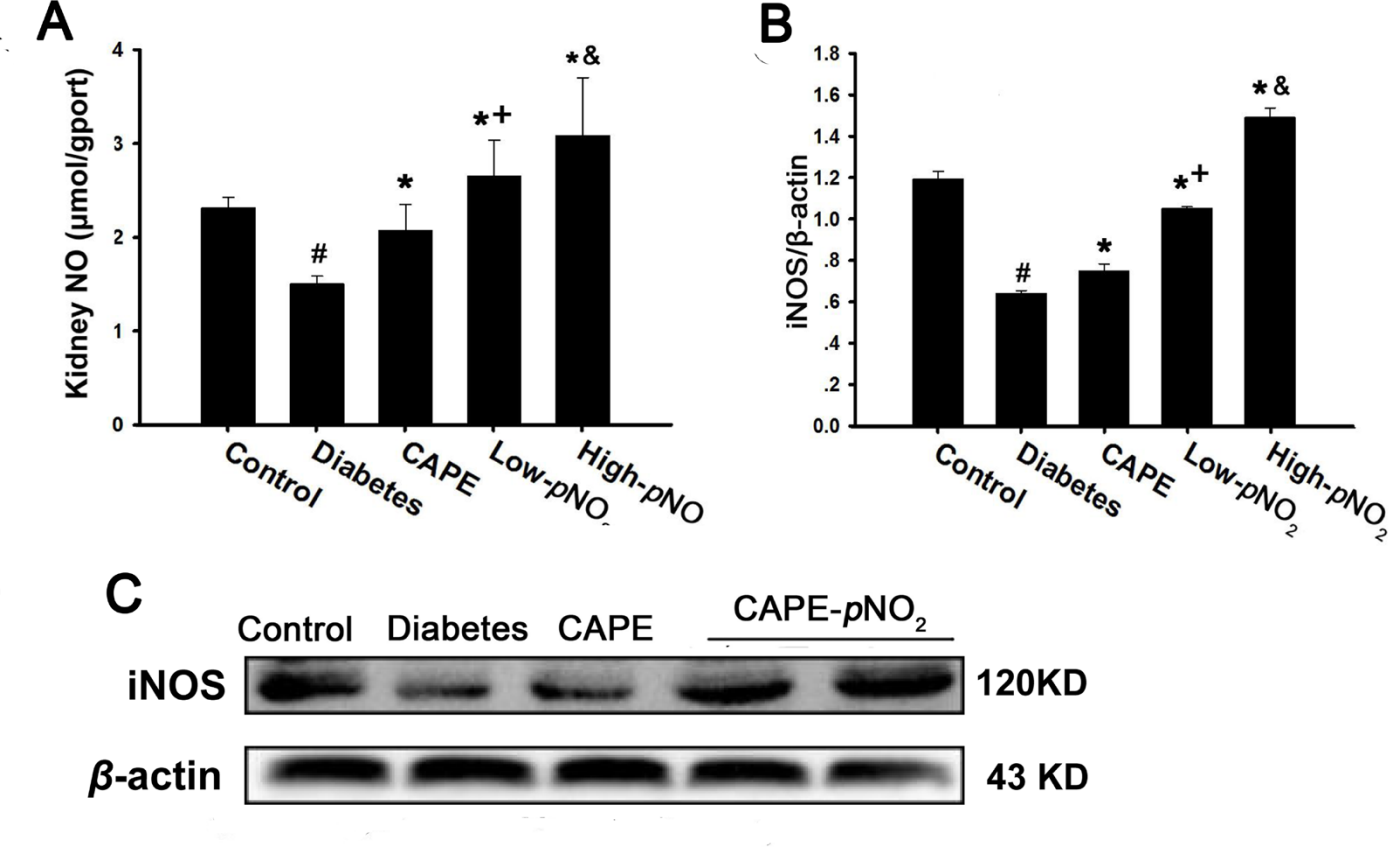
CAPE and CAPE-*p*NO_2_ increased NO content via regulating iNOS expression in diabetic mice (**A**) the NO level; (**B**, **C**) the iNOS protein expression by CAPE and CAPE-*p*NO_2_ treatment. Data are expressed as mean ± SD, *n* ≥ 6. ^#^*p* < 0.05 vs. control group; ^*^p < 0.05 vs. diabetes group; ^+^*p* < 0.05 vs. CAPE group; ^&^*p* < 0.05 vs. Low-*p*NO_2_ group. Diabetes: the STZ-induced diabetic mice; CAPE: the STZ-induced diabetic mice treated with CAPE (20 μmol/kg/day); Low-*p*NO_2_: the STZ-induced diabetic mice treated with CAPE-*p*NO_2_ (20 μmol/kg/day); High-*p*NO_2_: the STZ-induced diabetic mice treated with CAPE-*p*NO_2_ (40 μmol/kg/day).

As shown in Figure 7A, NO concentrations were suppressed remarkably in diabetic mice (*p* < 0.05) but were increased after treatment with CAPE and CAPE-*p*NO_2_; the effects of CAPE-*p*NO_2_ were better than those of CAPE (*p* < 0.05). Similarly, the results shown in Figure 7B and 7C indicated that iNOS expression was inhibited in the diabetes group (*p* < 0.05), whereas CAPE and CAPE-*p*NO_2_ up-regulated iNOS expression; again, CAPE-*p*NO_2_ had better effects than CAPE (*p* < 0.05). The effects of CAPE-*p*NO_2_ on NO and iNOS were concentration-dependent.

### CAPE and CAPE-*p*NO_2_ suppressed HG-induced GMC proliferation

**Figure 8:**
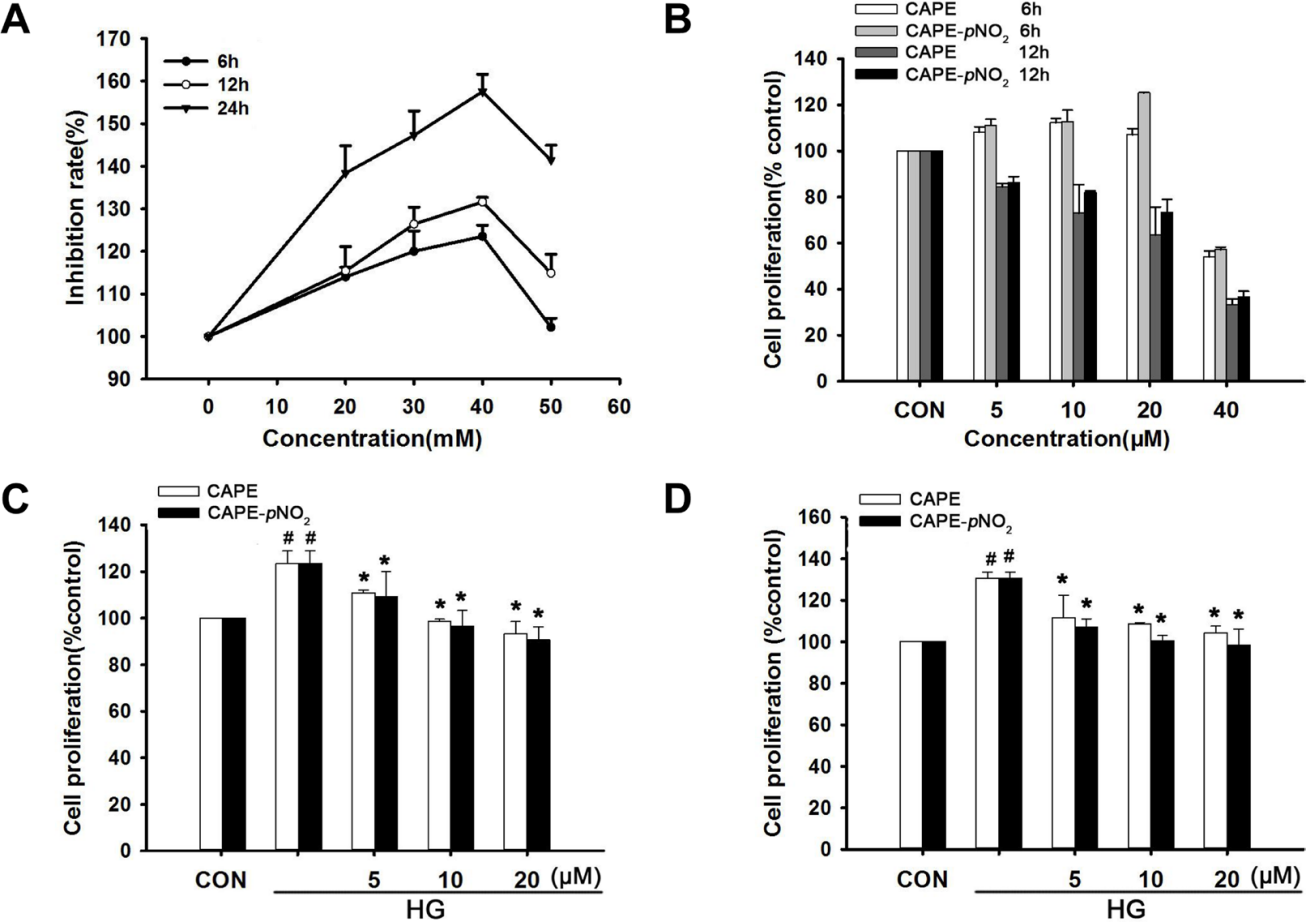
CAPE and CAPE-*p*NO_2_ suppressed HG-induced GMC proliferation (**A**) Glomerular mesangial cells (GMCs) were induced by various concentration D-glucose from 6 h to 24 h. (**B**) GMCs were treated with CAPE and CAPE-*p*NO2 at the concentration of 5, 10, 20, 40 μΜ from 6h to 12 h in normal D-glucose. (**C**, **D**) GMCs were treated with CAPE and CAPE-*p*NO_2_ at the concentration of 5, 10, 20, μΜ in 30 mΜ D-glucose for 3 h and 6 h. Data are expressed as mean ± SD, *n* = 3 per group. ^#^*p* < 0.05 vs CON group; ^*^p < 0.05 vs HG group. ^+^*p* < 0.05 vs CAPE group. CON group: GMCs kept in DMEM/F12 with 5.6 mM D-glucose. HG group: GMCs kept in DMEM/F12 with 30 mM D-glucose. CAPE and CAPE-*p*NO_2_ group: treated with 5, 10, 20 μΜ after induced by 30 mM D-glucose, respectively.

As shown in Figure 8A, an appropriate concentration of D-glucose (20–40 mM) promoted cell proliferation; however, cell proliferation was suppressed when the D-glucose concentration reached 50 mM. In addition, the cells presented with altered morphology when the concentration reached 40 mM. The cell proliferation induced by 30 mM D-glucose was 120%, 126% and 147% from 6 h to 24 h. Therefore, incubation with 30 mM D-glucose for 24 h was selected as the best condition to imitate hyperglycemia *in vitro*.

GMC proliferation was not changed obviously under normal glucose conditions and 20 μΜ CAPE or CAPE-*p*NO_2_ treatment for 6 h as shown in Figure 8B. CAPE and CAPE-*p*NO_2_ inhibited HG-induced GMC proliferation (*p* < 0.05), which occurred in a concentration and time-dependent manner, and CAPE-*p*NO_2_ was more effective than CAPE as shown in Figure 8C and 8D.

### CAPE and CAPE-*p*NO_2_ arrested HG-induced cell cycle progression

**Figure 9:**
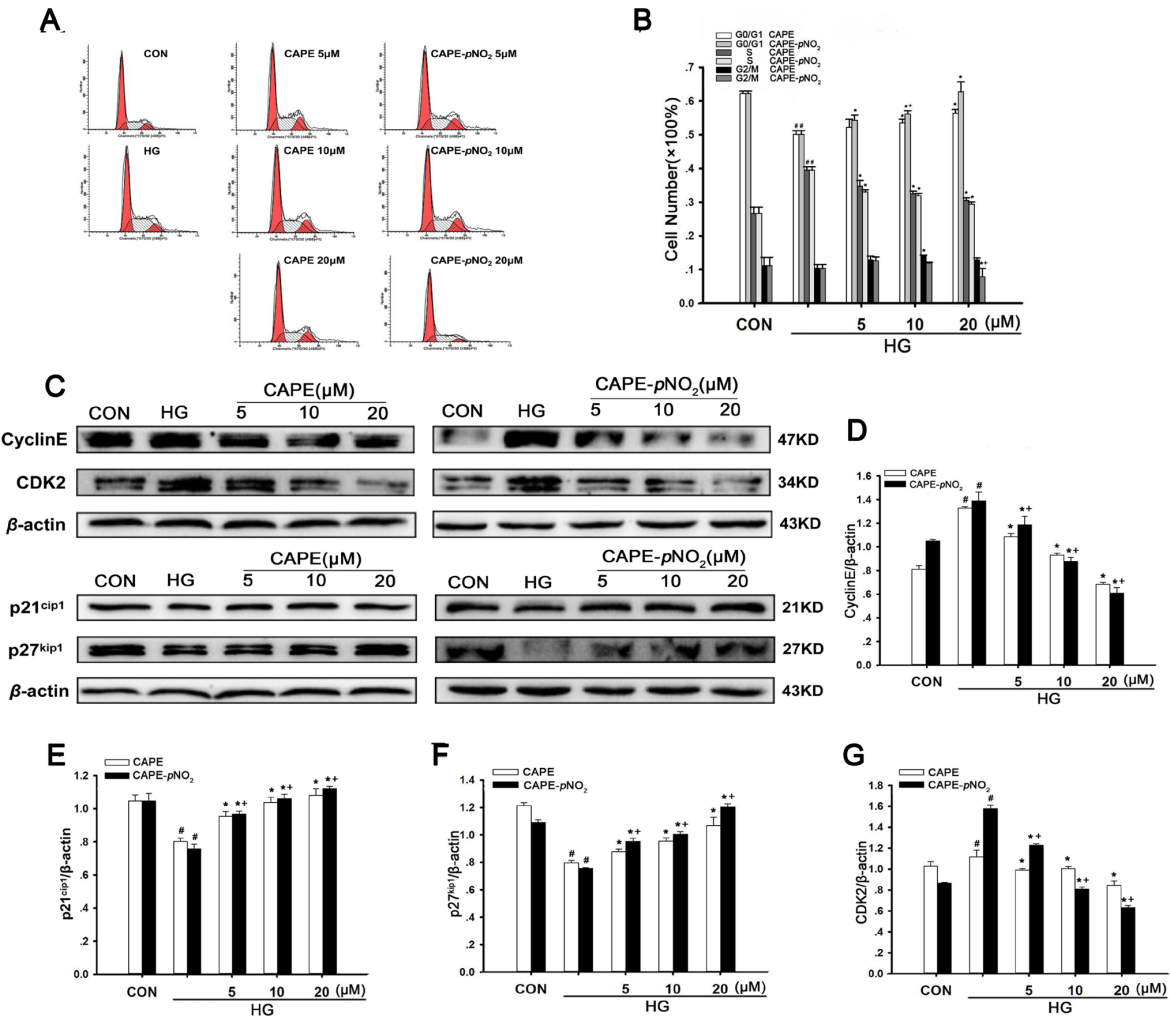
CAPE and CAPE-*p*NO_2_ arrested HG-induced cell cycle progression (**A**, **B**) Change of cell cycle distribution induced by CAPE and CAPE-*p*NO_2_ was investigate by flow cytometry and the percentage of cell in G0/G1, S and G2/M phase was calculated by a Multicycle software. (**C–G**) The expressions of CDK2, CyclinE, p21^cip1^ and p27^kip1^ were assessed by western blot analysis. Data are expressed as mean ± SD, *n* = 3 per group. ^#^*p* < 0.05 vs CON group; ^*^p < 0.05 vs HG group. ^+^*p* < 0.05 vs CAPE group. CON group: GMCs kept in DMEM/F12 with 5.6 mM D-glucose. HG group: GMCs kept in DMEM/F12 with 30 mM D-glucose. CAPE and CAPE-*p*NO_2_ group: treated with 5, 10, 20 μΜ after induced by 30 mM D-glucose, respectively.

Under conditions of hyperglycemia, the proportion of cells in the G0/G1 phase decreased from 62.2% to 50.1% compared with the control group. However, treatment with 20 μΜ CAPE and CAPE-*p*NO_2_ increased the proportion of cells in the G0/G1 phase to 56.5% and 62.3%, and the proportion of cells in the G2/M phase was barely altered. The results proved that the cell cycle from the G1 to S phase was interrupted by CAPE and CAPE-*p*NO_2_ treatment (Figure 9A, 9B).

Furthermore, we examined cell cycle-related proteins. As shown in Figure 9C–9G, HG significantly decreased the expression levels of p21^Cip1^ and p27^Kip1^ (*p* < 0.05) and increased the expression levels of cyclin E and Cdk2 (*p* < 0.05). However, CAPE and CAPE-*p*NO_2_ treatment up-regulated 1.3-, 1.5-fold in p21^Cip1^ and 1.3-, 1.6-fold in p27^Kip1^ expression (*p* < 0.05) but down-regulated 0.8-, 0.4-fold in Cdk2 and 0.5-, 0.4-fold in cyclin E expression (*p* < 0.05) in HG-induced GMCs at 20 μΜ. In addition, the effects of these compounds occurred in a concentration-dependent manner. These results suggest that CAPE and CAPE-*p*NO_2_ can suppress HG-induced cell cycle progression through inhibiting cell cycle-related proteins, and CAPE-*p*NO_2_ was much more effective than CAPE (*p* < 0.05).

### Effects of CAPE and CAPE-*p*NO_2_ treatment on oxidative stress and ECM expression in HG-induced GMCs

**Figure 10:**
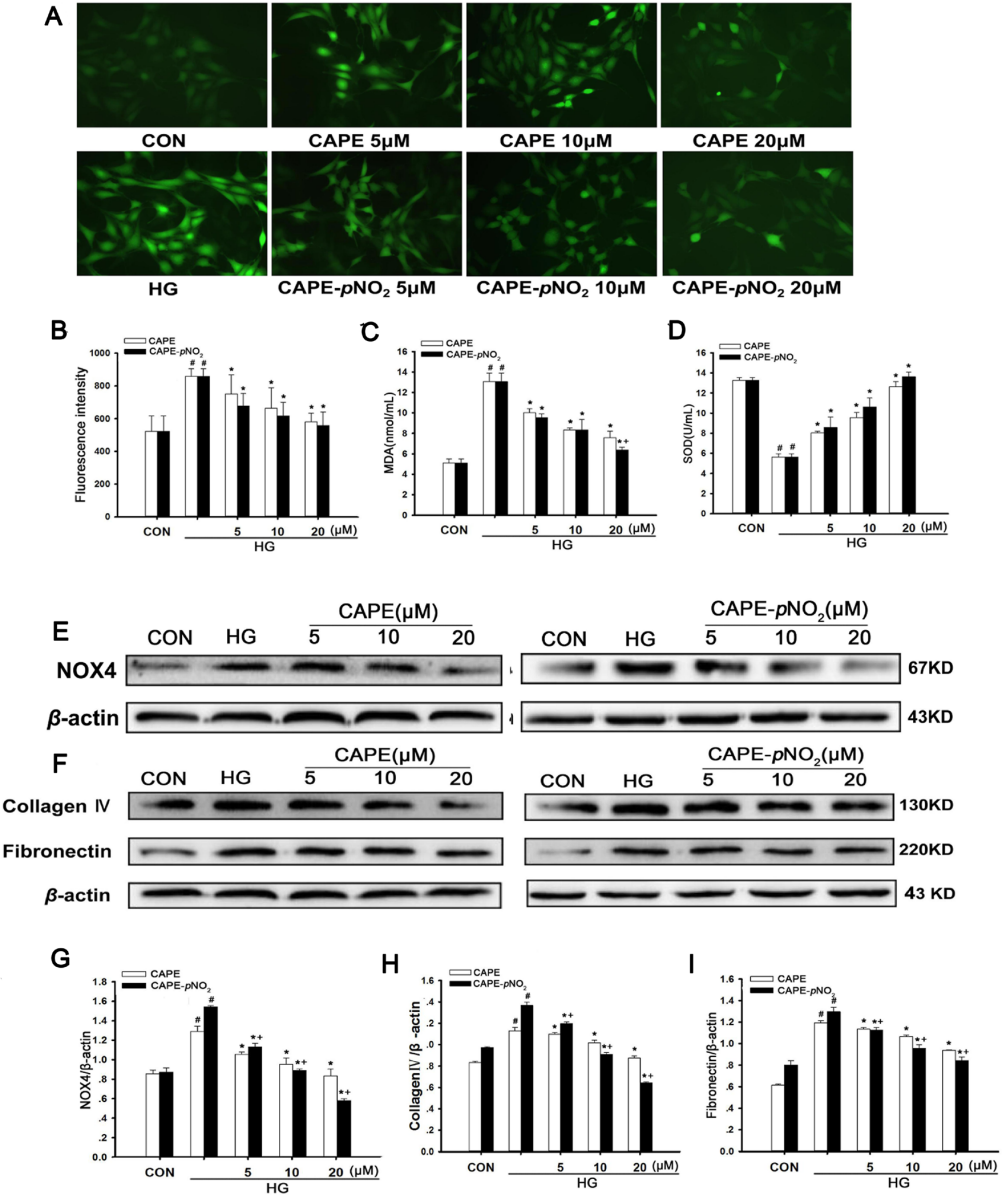
Effects of CAPE and CAPE-*p*NO_2_ treatment on oxidative stress and ECM expression in HG-induced GMCs (**A**, **B**) the intracellular ROS level in GMCs was detected by 2′,7′-dichlorodi-hydrofluorescein diacetate (DCFH-DA) and observed by a fluorescence microscope or a fluorospectrophotometer at excitation/emission maxima of 485/525 nm. (**C**, **D**) the intracellular MDA and SOD levels in GMCs. (**E**, **G**) the expression of NOX4 was assessed by western blot analysis. (**F**, **H**, **I**) The expressions of collagen IV and fibronectin were assessed by western blot analysis. Data are expressed as mean ± SD, *n* = 3 per group. ^#^*p* < 0.05 vs CON group; ^*^p < 0.05 vs HG group. ^+^*p* < 0.05 vs CAPE group. CON group: GMCs kept in DMEM/F12 with 5.6 mM D-glucose. HG group: GMCs kept in DMEM/F12 with 30 mM D-glucose. CAPE and CAPE-*p*NO_2_ group: treated with 5, 10, 20 μΜ after induced by 30 mM D-glucose, respectively.

The effects of CAPE and CAPE-*p*NO_2_ on oxidative stress *in vitro* are shown in Figure 10A and 10B. There was a significant increase in intracellular ROS generation in HG-induced GMCs (*p* < 0.05), and treatment with CAPE and CAPE-*p*NO_2_ decreased the ROS levels (*p* < 0.05). Moreover, as shown in Figure 10C and 10D, the activity of SOD was increased, and the MDA levels were decreased after treatment with CAPE and CAPE-*p*NO_2_ (*p* < 0.05). Furthermore, the expression of NOX4 was suppressed 0.6-, 0.4-fold by CAPE and CAPE-*p*NO_2_ treatment at 20 μΜ compared with HG-induced GMCs (*p* < 0.05) as shown in Figure 10E and 10G. These results indicate that CAPE and CAPE-*p*NO_2_ attenuated oxidative stress, and the effects of CAPE-*p*NO_2_ were better than those of CAPE (*p* < 0.05).

In addition, the expression levels of fibronectin and collagen IV in GMCs were markedly increased under conditions of hyperglycemia (*p* < 0.05) (Figure 10F, and 10I). Treatment with CAPE and CAPE-*p*NO_2_ decreased 0.8- and 0.6-fold in fibronectin and 0.6- and 0.5-fold in collagen IV at 20 μΜ compared to the HG-induced group (*p* < 0.05); these results were similar to those of the animal experiment and showed that CAPE-*p*NO_2_ had better effects than CAPE (*p* < 0.05), and occurred in a concentration-dependent manner.

### CAPE and CAPE-*p*NO_2_ treatment suppressed TGF-β1 expression via the NF-κB pathway in HG-induced GMCs

**Figure 11:**
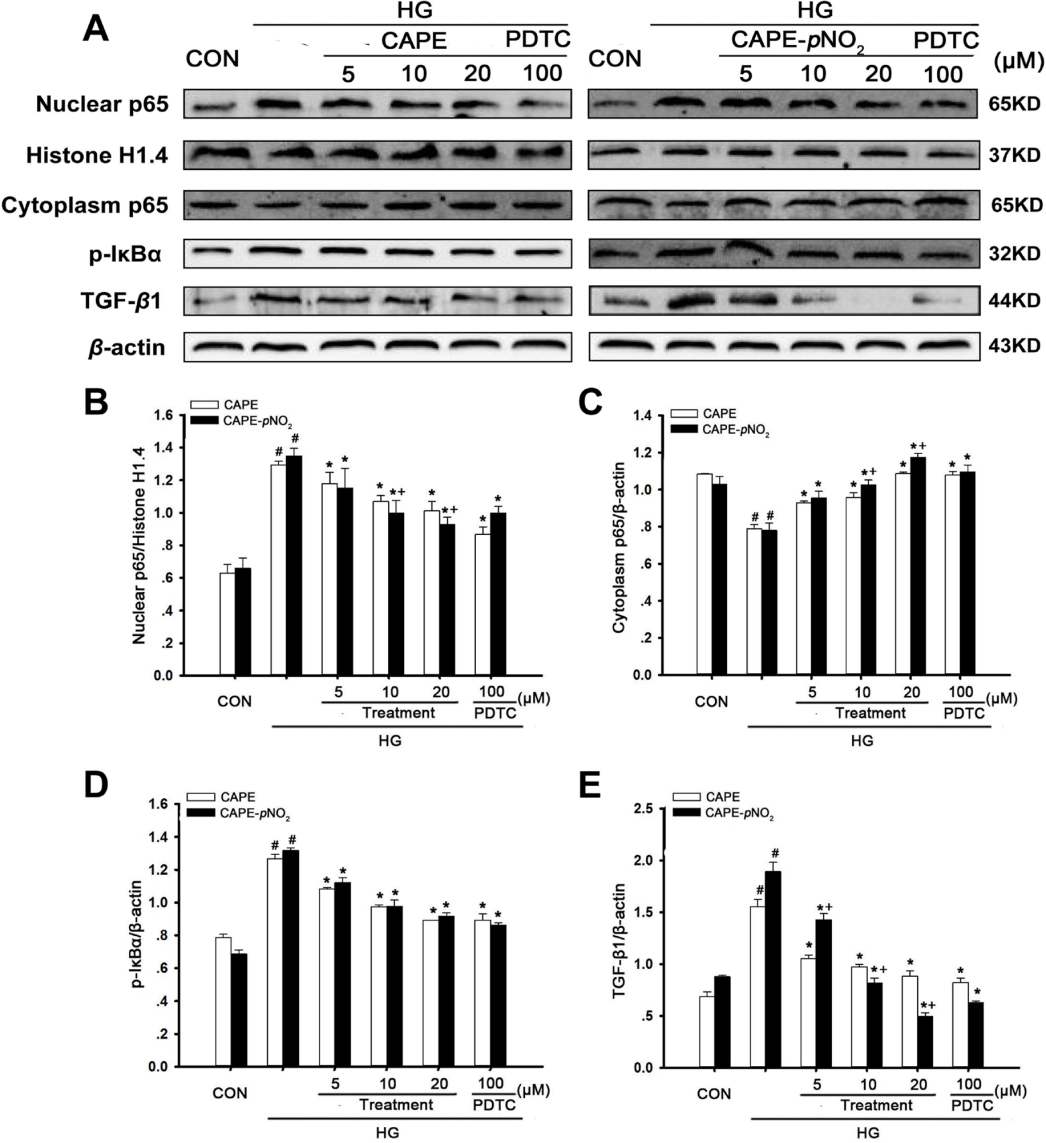
CAPE and CAPE-*p*NO_2_ treatment suppressed TGF-β1 expression via the NF-κB pathway in HG-induced GMCs (**A**–**E**) the expressions of p65 in nuclear and cytoplasm, p-IκBα, TGF-β1 were assessed by western blot analysis. Data are expressed as mean ± SD, *n* = 3 per group. ^#^*p* < 0.05 vs CON group; ^*^p < 0.05 vs HG group. ^+^*p* < 0.05 vs CAPE group. CON group: GMCs kept in DMEM/F12 with 5.6 mM D-glucose. HG group: GMCs kept in DMEM/F12 with 30 mM D-glucose. CAPE and CAPE-*p*NO_2_ group: treated with 5, 10, 20 μΜ after induced by 30 mM D-glucose, respectively. PDTC group: treated with 100 μΜ after induced by 30 mM D-glucose.

As shown in Figure 11A–11E, HG treatment for 24 h markedly increased cytoplasm p65 levels, as well as p-IκBα and TGF-β1 levels, and decreased nuclear p65 expression levels (*p* < 0.05). However, CAPE and CAPE-*p*NO_2_ significantly increased 1.4- and 1.5-fold in cytoplasm p65 expression and decreased 0.8- and 0.7-fold in nuclear p65; as well as p-IκBα was 0.7- and 0.7-fold and TGF-β1was 0.6- and 0.3-fold at 20 μΜ, compared with the HG-induced GMCs, which had similar effects to those of caused by 100 μM PDTC treatment. CAPE-*p*NO_2_ had better effects than CAPE on p65 and TGF-β1 expression, but there were no differences in the effects on p-IκBα expression.

### Effects of CAPE and CAPE-*p*NO_2_ treatment on iNOS expression in HG-induced GMCs

**Figure 12:**
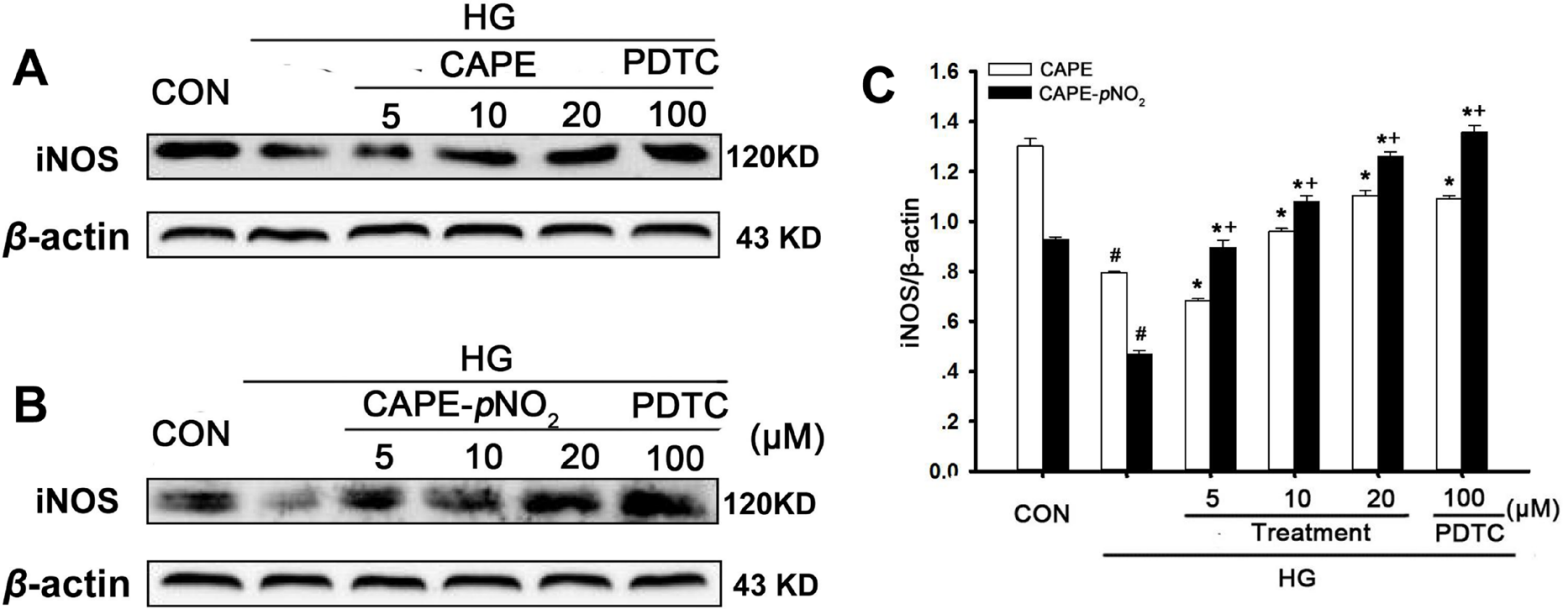
Effects of CAPE and CAPE-*p*NO_2_ treatment on iNOS expression in HG-induced GMCs (**A**–**C**) the expression of iNOS protein was assessed by western blot analysis. Data are expressed as mean ± SD, *n* = 3 per group. ^#^*p* < 0.05 vs CON group; ^*^p < 0.05 vs HG group. ^+^*p* < 0.05 vs CAPE group. CON group: GMCs kept in DMEM/F12 with 5.6 mM D-glucose. HG group: GMCs kept in DMEM/F12 with 30 mM D-glucose. CAPE and CAPE-*p*NO_2_ group: treated with 5, 10, 20 μΜ after induced by 30 mM D-glucose, respectively. PDTC group: treated with 100 μΜ after induced by 30 mM D-glucose.

The effects of CAPE-*p*NO_2_ on the iNOS expression in GMCs were similar to those in kidney tissues. As shown in Figure 12A–12C, iNOS expression in GMCs in the HG-induced group was decreased (*p* < 0.05). Treatment with PDTC up-regulated iNOS expression, and CAPE and CAPE-*p*NO_2_ had similar effects, which increased 1.4- and 2.7-fold in iNOS expression at 20 μM. CAPE-*p*NO_2_ had better effects than CAPE (*p* < 0.05), and these effects occurred in a positive concentration-dependent manner.

## DISCUSSION

Diabetes mellitus is one of the most widespread metabolic diseases in the world; chronic hyperglycemia in diabetes leads to dysfunction in various tissues, especially in the eyes, kidneys, heart, blood vessels and nerves [44]. DN is the most important complication of diabetes, and it greatly affects patient quality of life. In recent years, therapeutic strategies for DN are mainly focused on controlling blood pressure and blood glucose levels, diet therapy and organ transplants, but these strategies do not stop the development of kidney disease and are expensive. It has been reported that CAPE and caffeic acid phenylethyl amide exhibit significant potential as anti-diabetic and liver-protective agents in STZ-induced diabetic rats; these compounds could protect the renal system from ischemia-reperfusion injury via anti-fibrotic mechanisms [40, 45]. CAPE-*p*NO_2_, a derivative of CAPE, was synthesized and demonstrated better effects than CAPE on acute myocardial ischemia-reperfusion injury through anti-oxidative stress, anti-inflammatory and anti-apoptotic activities in rats in our previous research [40].

In the present experiment, CAPE-*p*NO_2_ significantly increased body weights and decrease FBG levels in STZ-induced diabetic mice; it also reversed the changes in biochemical indexes, such as 24-h Alb, sCr, BUN, TG and TC levels, which reflect the function of the kidneys [46]. These results imply that CAPE-*p*NO_2_ can ameliorate the symptoms of diabetes mellitus and DN and improve renal function in STZ-induced diabetic mice. In addition, in the *in vitro* assay with GMCs, CAPE-*p*NO_2_ inhibited proliferation in HG-induced GMCs, arrested HG-induced cell cycle progression, suppressed ROS generation, and also significantly decreased NOX4 and ECM protein expression.

GMCs maintain the organizational structure and physiological functions of the kidney, and abnormal GMC proliferation plays an important role in the development of DN [47]. It has been reported that p21^Cip1^, p27^Kip1^, Cdks and cyclin E are involved in the G1-S phases of the cell cycle and that Cdks combine with cyclin E to promote the cell cycle, which contributes to proliferation, differentiation and aging in cells; in addition, high glucose conditions down-regulate p21^Cip1^ and p27^Kip1^ expression in GMCs [47–51]. Our data indicate that CAPE and CAPE-*p*NO_2_ can reverse HG-induced cell cycle-related proteins to arrest the cell cycle at G1-S, which could cause the suppression of HG-induced GMC proliferation and ECM accumulation.

It is well known that imbalances in the oxidative stress defense system could damage the kidney. In this system, SOD is an enzyme that scavenges reactive oxygen radicals and converts harmful super oxygen radicals into hydrogen peroxide to protect against kidney damage from free radicals, whereas MDA is a degradation product of oxygen-derived free radicals and lipid oxidation that causes cytotoxicity. The interaction of oxidative stress generation with biomolecules, such as lipids, proteins and DNA, can activate a series of cell signaling pathways, leading to severe injury and dysfunction in the kidney. In addition, under long-term hyperglycemia conditions, the balance of anti-oxidative/oxidative stress is destroyed and ROS are over produced [52–54]. NADPH oxidation is a major source of ROS production, and the NADPH oxidase 4 (NOX4) is the major active subgroup of NADPH oxidase, and high levels of ROS was important in the progression of diabetic nephropathy [27, 55–59]. In anti-oxidative stress, CAPE and CAPE-*p*NO_2_ dramatically attenuated MDA levels and increased SOD activity in STZ-induced diabetic mice and HG-induced GMCs. In addition, ROS generation was decreased by CAPE and CAPE-*p*NO_2_ treatment in HG-induced GMCs. Our results show that CAPE-*p*NO_2_ can maintain balance between the oxidative stress defense systems in diabetic mice and HG-induced GMCs and that CAPE-*p*NO_2_ has better effects on anti-oxidative stress *in vivo* and *in vitro*.

**Figure 13:**
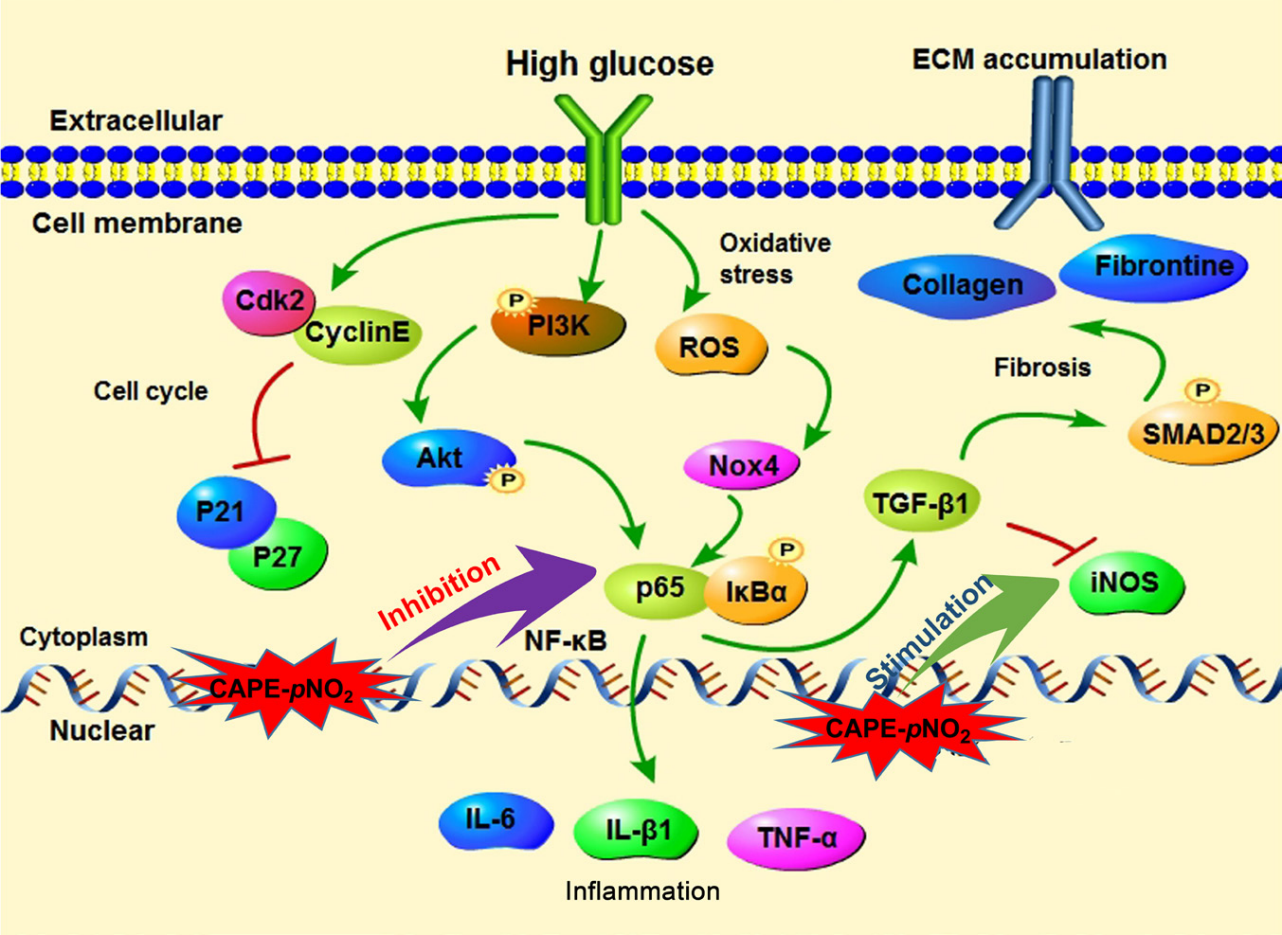
The possible mechanism of CAPE and CAPE-*p*NO_2_ on DN (**A**) The CAPE and CAPE-*p*NO_2_ regulated the Akt/NF-κB/iNOS pathway to ameliorate oxidative stress, fibrosis and inflammation in diabetic mice. (**B**) The CAPE and CAPE-*p*NO_2_ suppressed the HG-induced GMCs proliferation via arresting the cell cycle and reducing the ECM accumulation via regulating the TGF-β1.

Imbalance in the oxidative stress defense systems could regulate sensitive cytokines. ROS promotes NF-κB transcription though activating IkBα kinase, and the p65 subunit is transferred into the nucleus and binds to the specific promoter sequence of target genes, such as TNF-α, IL-1β, and IL-6, which results in inflammatory injury in the kidneys. Moreover, Akt is an upstream element of the NF-κB pathway that can mediate the development of inflammatory diseases and promote the transcription of NF-κB [3, 60, 61]. In our experiment, we showed that CAPE and CAPE-*p*NO_2_ inhibit the Akt/NF-κB pathway to regulate the release of inflammatory cytokines in diabetic mice (Figure 13).

Activated NF-κB that translocates into the nucleus can also induce the expression of TGF-β1, which is a key fibrotic cytokine, and mediate ECM accumulation via the TGF-β1/Smad pathway [55, 62–64]. The TGF-β1 promoter has been confirmed to contain a sequence located at 715 to 707 bp (AGGGACTT) where NF-κB binds to target TGF-β1 gene expression [65]. In addition, the Smad family is a classic downstream signal transduction molecule of TGF-β1, and the activated phosphorylation of Smad2 and Smad3 could promote the deposition of ECM proteins, which comprise collagen, elastin, laminin and fibronectin and are secreted mainly from GMCs [66, 67]. They contribute to renal fibrosis and have been recognized as part of the major pathogenic events in the progression of renal failure in DN [62, 68, 69]. Hence, the overexpression of TGF-β1 may be the main cause of glomerulosclerosis and tubulointerstitial fibrosis, and the inhibition of TGF-β1 could be beneficial for treating DN. *In vivo* experiments using HE and Masson staining showed that CAPE and CAPE-*p*NO_2_ prevented kidney fibrosis, which may be associated with the suppression of the TGF-β1/Smad pathway. *In vitro* experiments in which the NF-κB inhibitor PDTC was incubated with HG-induced GMCs were used to validate the relationship between NF-κB and ECM proteins. PDTC promoted p65 subunit transfer into the nucleus and binding to the specific promoter sequences of target genes. Our results showed the 100 μΜ PDTC suppressed the transcription of p65. CAPE and CAPE-*p*NO_2_ showed effects similar to PDTC, and CAPE-*p*NO_2_ was more efficient than CAPE. Therefore, CAPE and CAPE-*p*NO_2_ reduced ECM accumulation via regulating the TGF-β1, and the TGF-β1 expression was associated with the NF-κB pathway *in vitro* (Figure 13).

More importantly, NO is decreased in CKD; this contributes to cardiovascular events and further causes the progression of kidney damage [70, 20]. iNOS drives the production of NO, which affects many features *in vivo* and *in vitro*. For DN, we can infer that a relationship between NO and DN must exist. Moreover, it has been reported that TGF-β1 can suppress iNOS mRNA expression induced by LPS, IFN-γ, IL1-β, and various cytokines and can promote iNOS protein degradation via the ubiquitin-proteasome pathway. These data indicate that TGF-β1 control of the NO content produced by iNOS in pathological conditions is critical [71, 72]. Our results show that CAPE and CAPE-*p*NO_2_ can increase NO content via regulating iNOS expression in diabetic mice and up-regulating iNOS expression in HG-induced GMCs. It can be inferred that iNOS may be have a direct relationship with TGF-β1 and an indirection relationship with the NF-κB pathway (Figure 13); these may be key points that are worth exploring in DN.

In one word, CAPE-*p*NO_2_ possessed better activities in regulating of the Akt/NF-κB/iNOS pathway in STZ-induced diabetic mice. CAPE-*p*NO_2_ is a nitro derivative of CAPE, the reason of its stronger pharmacological activities may be nitro replacement changing its molecular properties. The nitro-group is an electron withdraw group, it could reduce the cloud density of benzene ring to change the bioactivities of CAPE-*p*NO_2_ probably [73], and this change is needs to be further verified.

## CONCLUSIONS

In summary, our data suggest that CAPE and CAPE-*p*NO_2_ can ameliorate DN by suppressing renal oxidative stress, inflammation and fibrosis in STZ-induced diabetic mice via the Akt/ NF-κB/ iNOS pathway; additionally, these compounds can inhibit cell proliferation and ECM accumulation via regulating TGF-β1expression in HG-induced GMCs. Furthermore, CAPE-*p*NO_2_ had stronger effects than CAPE. Our data imply that CAPE-*p*NO_2_ has kidney- protective effects against DN and can serve as a potential drug for treating DN. However, these possibilities still needed to be explored.

## MATERIALS AND METHODS

### Materials

CAPE and CAPE-*p*NO_2_ were synthesized as previously described in the literature [40] (purity >99.0%). Glomerular mesangial cells (GMCs) were purchased from the Cell Bank of the Chinese Academy of Sciences (Shanghai, China). STZ, trypsin, penicillin, streptomycin, dimethyl sulfoxide (DMSO), bovine serum albumin (BSA), 3-[4,5-dimethyl-2-thiazolyl] -2,5-diphenyl-2-tetrazolium bromide (MTT) and propidium iodide (PI) were purchased from Sigma Chemicals (St. Louis, MO, USA). A glucometer and blood glucose test strips were purchased from Sannuo (Guangxi, China). Blood urea nitrogen (BUN), serum creatinine (sCr), urine protein (UR), total cholesterol (TC), total triglyceride (TG), malondialdehyde (MDA), myeloperoxidase (MPO), nitric oxide (NO) and superoxide dismutase (SOD) assay kits were purchased from Nanjing Jiancheng Bioengineering Institute (Nanjing, China). Dulbecco’s modified Eagle’s medium (DMEM), F12 and fetal bovine serum (FBS) were purchased from Gibco/Invitrogen (Carlsbad, CA, USA). RNase A was acquired from Promega (Madison, WI). 2′,7′-dichlorofluorescein- diacetate (DCFH-DA), PDTC and Nuclear and Cytoplasmic Protein Extraction Kit were purchased from Beyotime Biotechnology (Shanghai, China). Antibodies against Akt, PI3K, p65, TGF-β1, α-SMA, Smad2/3, TNF-α, fibronectin, collagen IV, IL-6, IL-β, IκBα, CDK2, cyclin E, p21^Cip1^, p27^Kip1^, iNOS, p-Akt, p-PI3K, p-Smad2/3 and p-IκBα, histone H1.4 and β-actin and horseradish peroxidase (HRP)-conjugated goat anti-rabbit secondary antibody were purchased from Proteintech Group Inc (Wuhan, China).

### Animals and treatment

Eight-week-old Kunming male mice (18–22 g) were purchased from the Experimental Animal Center of Chongqing Medical University (Chongqing, China SCXK (YU) 2016–0002). They were housed under a 12 h light/dark cycle at 22°C with access to food and water. All animal procedures were conducted according to the China Animal Welfare Legislation and approved by the Committee for the Care and Use of Laboratory Animals at Southwest University. Fifty mice were randomly divided into two groups: control group and diabetes group. The diabetes group had 40 mice, which were intraperitoneally injected with STZ dissolved in 10 mM citrate buffer (pH 4.5) at 50 mg/kg body weight for 5 consecutive days. The control group was injected with the same volume of citrate buffer.

Blood samples were obtained from the tails of the mice, and fasting blood glucose (FBG) levels were measured with a glucometer. Mice with the FBG levels above 11.1 mmol/L were considered to diabetes; these mice were then divided randomly into four groups: 1) the diabetes group, mice treated with normal saline, 2) the CAPE group, mice treated with CAPE (20 μmol/kg/day), 3) the low CAPE-*p*NO_2_ group, mice treated with CAPE-*p*NO_2_ (20 μmol/kg/day), and 4) the high CAPE-*p*NO_2_ group, mice treated with CAPE-*p*NO_2_ (40 μmol/kg/day). CAPE and CAPE-*p*NO_2_ were dissolved in 0.01% DMSO and intraperitoneally injected for 8 weeks. FBG levels were tested once a week using tail-vein blood samples, and the mouse body weights were recorded every three days for 8 weeks. On the last day of the 8th week, the mice were housed in metabolic cages to collect urine to test 24-h albumin excretion rates. After the above experiments were finished, all mice were fasted for 12 h and then euthanized; then, the kidneys and blood samples were collected for the following experiments.

### Biochemical indexes

The kidneys were weighed to calculate the kidney-to-body weight ratio (ratio = body weight / kidney weight × 100%). The urine was centrifuged at 2000 rpm for 10 min at 4°C to isolate the supernatant. In addition, the blood samples were centrifuged at 3000 rpm for 15 min at 4°C to separate the serum. The levels of cholesterol, triglycerides, blood urea nitrogen and creatinine in the serum and 24-h albumin excretion were tested by kits according to the respective manufacturer’s protocol.

### HE, PAS, Masson and immunohistochemical staining of the kidneys

The kidneys were fixed in a 4% paraformaldehyde solution for 48 h and then embedded in paraffin. The 5 μm-thick serial sections cut from paraffin blocks were stained with HE, PAS and Masson trichrome solutions. Images of these stained sections were obtained using a light microscope.

For immunohistochemistry experiments, 5 μm-thick sections were deparaffinized, hydrated and immersed in 10 mM citric acid (pH 6.0); next, the endogenous peroxidase activity was blocked with 3% H_2_O_2_. Then, the kidney sections were incubated with primary antibodies (anti-TNF-α, 1:200) at 4°C overnight. The next day, the kidney sections were incubated with HRP-conjugated goat anti-rabbit IgG secondary antibody for 2 h at room temperature and washed with PBS (3 × 5 min). After that, the sections were stained with the diaminobenzidine (DAB) and hematoxylin. Finally, images of the sections were obtained with a light microscope, and the positive-stained areas were measured and quantified using IPP, which presented the expression levels of the corresponding protein.

### Cell culture and treatment

GMCs were grown in DMEM/F-12(3:1) supplemented with 5% FBS, 100 U/mL penicillin and 100 μg/mL streptomycin at 37°C in a humidified incubator (5% CO2, 95% air). The cells were synchronized in serum-free medium for 24 h and then divided into the following groups: (a) control, DMEM/F-12 medium with normal D-glucose (5.6 mM), (b) high D-glucose, DMEM/F-12 medium with high D-glucose (30 mM), (c-e) high D-glucose + treatment, DMEM/F-12 medium with high D-glucose and treatment with CAPE or CAPE-*p*NO_2_ (5, 10 or 20 μM), (f) high D-glucose + PDTC, DMEM/F-12 medium with high D-glucose and treatment with PDTC (100 μM).

### Cell proliferation assay

The effects of CAPE and CAPE-*p*NO_2_ on cell proliferation were measured by MTT assay. GMCs were seeded at a density of 3 × 10^3^ cells per well in 96-well plates and incubated with normal D-glucose or high D-glucose concentrations in the presence or absence of CAPE or CAPE-*p*NO_2_ (5, 10 or 20 μM) for 3 h or 6 h. A total of 10 μL of MTT (5 mg/mL) was then added to each well for another 4 h at 37°C. After incubation, 150 μL of DMSO was added and slowly shaken until the tetrazolium dye dissolved. The amount of formazan dissolved in DMSO was measured by a plate reader (Bio Tek) at 490 nm.

### Reactive oxygen species (ROS) evaluation in GMCs

Intracellular ROS levels were measured using DCFH-DA. GMCs were seeded in 6-well plates and treated with CAPE or CAPE-*p*NO_2_ at 5, 10 or 20 μΜ for 6 h. After treatment, GMCs were washed with FBS-free culture medium three times and incubated with 10 mM DCFH-DA in the dark for 30 min at 37°C ; then, the cells were washed with FBS-free culture medium, and the cells were observed under a fluorescence microscope or were measured by a fluorospectrophotometer at an excitation/emission of 485/525 nm.

### Cell cycle analysis

The cell cycle distribution was evaluated by flow cytometry. After treatment with CAPE or CAPE-*p*NO_2_ at 5, 10 or 20 μΜ for 6 h, the cells were collected and fixed in 70% ice-cold ethanol at 4°C overnight. The next day, the cells were incubated with 100 μL of RNase A and 400 μL of PI at room temperature for 30 min without light. Then, a FACS Calibur flow cytometer (Becton-Dickinson) equipped with a 488-nm argon laser was used for detection, and the cell cycle distribution was analyzed by Cell Quest software and ModiFit according to the manufacturer’s instructions.

### SOD, MDA, MPO and NO evaluation in mice and GMCs

Serum was separated from the blood samples. The kidneys were rinsed with 0.9% saline and homogenized at 10,000 rpm in an ice bath for 5 min to prepare 10% homogenates; the samples were subsequently centrifuged 10,000 rpm at 4°C for 10 min to collect the supernatants. After GMCs were treated with CAPE or CAPE-*p*NO_2_ (5, 10 or 20 μM) for 6 h, the cells were washed with PBS three times and collected; then, the cells were lysed with RIPA buffer and 1 mM PMSF for 30 min, followed by centrifugation at 10,000 rpm and 4°C for 10 min to separate the supernatants.

Serum samples and the supernatants from the kidneys and GMCs were collected to determine the SOD and MDA levels. The MPO levels in the serum samples and the kidneys and the NO levels in the kidneys were determined according to the respective manufacturer’s protocol.

### Western blotting assay

The kidneys were homogenized in RIPA lysis buffer and then centrifuged for 15 min at 10,000 rpm to extract the proteins. After the GMCs were treated with CAPE or CAPE-*p*NO_2_ at 5, 10 or 20 μΜ for 6 h, the cells were washed three times with cold PBS, and the nuclear and cytoplasmic proteins were extracted by a commercially available assay. Then, the cells were centrifuged for 15 min at 10,000 rpm, and the cytoplasm and nuclear proteins were collected for the next experiment. The protein samples were boiled for 10 min at 95°C and resolved using SDS-PAGE. Proteins on the gel were transferred to PVDF membranes, which were soaked in methanol for 15 min. Next, 5% skim milk prepared with PBST (0.1% Tween-20 and 99.9% PBS) was used to block the membrane for 1.5 h. Following this, the blots were incubated with primary antibodies for Akt, p-Akt, PI3K, p-PI3K, p65, IκBα, p-IκBα, IL-6, IL-β, TNF-α, TGF-β1, p-Smad2/3, Smad2/3, α-SMA, fibronectin, collagen IV, p27^kip1^, p21^cip1^, cyclin E, CDK2, iNOS, histone H1.4 and β-actin overnight at 4°C. The corresponding HRP-linked secondary antibody was incubated with the blots for 1.5 h after washing with PBST three times. The blots were detected by ECL (enhanced chemiluminescence) and quantified by Quantity One.

### Statistical analysis

All experiments were performed at least in triplicate. Data were processed using SPSS 16.0 software (SPSS, Inc., Chicago, IL, USA) and are presented as the mean ± SD. Statistical analyses of the data were performed by one-way ANOVA using post hoc multiple comparisons; *p* < 0.05 was considered a significant difference.

## Abbreviations

BUN: blood urea nitrogen
CAPE: caffeic acid phenethyl ester
CAPE-*p*NO_2_: caffeic acid para-nitro phenethyl ester
CKD: chronic kidney disease
DCFH-DA: 2′,7′-dichlorofluorescein-diacetate
DMEM: Dulbecco’s modified eagle medium
DMSO: dimethyl sulfoxide
DN: Diabetic Nephropathy
ECM: extracellular matrix
ESRD: end-stage renal disease
FBG: Fasting blood glucose
FBS: fetal bovine serum
GMCs: glomerular mesangial cells
HG: high D-glucose
iNOS: inducible nitric oxide synthase
Il-β: interleukin-1β
Il-6: interleukin-6
MDA: malondialdehyde
MPO: myeloperoxidase
MTT: 3-[4, 5-dimethyl-2-thiazolyl]-2,5-diphenyl-2-tetrazolium bromide
NO: Nitric oxide
PDTC: Ammonium pyrrolidinedithiocarbamate
PI: propidium iodide
ROS: reactive oxygen specie
STZ: streptozotocin
NOX4: NADPH oxidases 4
sCr: serum creatinine
SOD: superoxide dismutase
TC: Total cholesterol
TG: Total triglyceride
TNF-α: tumor necrosis factor-α
24-Alb: 24- albumin
